# Hypertension and Obesity: Risk Factors for Thyroid Disease

**DOI:** 10.3389/fendo.2022.939367

**Published:** 2022-07-18

**Authors:** Feng Liu, Xinyu Zhang

**Affiliations:** ^1^ West China Hospital, Sichuan University, Chengdu, China; ^2^ Department of Data Science and AI, Faculty of Information Technology, Monash University, Melbourne, VIC, Australia

**Keywords:** data mining, association rule mining, thyroid disease pathogenesis, risk factors, machine learning

## Abstract

Thyroid disease instances have rapidly increased in the past few decades; however, the cause of the disease remains unclear. Understanding the pathogenesis of thyroid disease will potentially reduce morbidity and mortality rates. Currently, the identified risk factors from existing studies are controversial as they were determined through qualitative analysis and were not further confirmed by quantitative implementations. Association rule mining, as a subset of data mining techniques, is dedicated to revealing underlying correlations among multiple attributes from a complex heterogeneous dataset, making it suitable for thyroid disease pathogenesis identification. This study adopts two association rule mining algorithms (i.e., Apriori and FP-Growth Tree) to identify risk factors correlated with thyroid disease. Extensive experiments were conducted to reach impartial findings with respect to knowledge discovery through two independent digital health datasets. The findings confirmed that gender, hypertension, and obesity are positively related to thyroid disease development. The history of I_131_ treatment and Triiodothyronine level can be potential factors for evaluating subsequent thyroid disease.

## 1 Introduction

Thyroid disease instances are rapidly increasing worldwide, and thyroid cancer is even ranked as the fifth most commonly diagnosed disease among females in the United States (1). Based on the statistics conducted by (2), there will be an estimated 43,800 new cases of thyroid cancer in the US by 2022. With the progressive diagnostic rates, understanding the pathogenesis of thyroid disease will potentially allow avoiding risk factors, leading to mitigated morbidity and mortality rates.

As of now, researchers are struggling to confirm the leading cause of thyroid disease. A few studies applied systematic literature analysis to identify potential risk factors correlated with the disease. The identified factors include radiation, depression, obesity, hormonal factors, and gene heredity (3). Nevertheless, many factors are still controversial and cannot be verified solely by relying on qualitative analysis. Medical datasets are complex in dimension as many factors interwove one and the other, making the identification of thyroid disease pathogenesis a challenging task (4). Additionally, the implementation of quantitative analysis has been considerably ignored in existing works; this again aggravates the limited reliability of the identified factors.

Association rule mining (ARM) is tailored to find and describe hidden associations among multiple attributes (5). It is efficient in dealing with complex, sensitive, and heterogeneous medical sets (6), making it fitting for investigating the potential risk factors correlated with thyroid disease. ARM techniques have been proven efficient in extracting knowledge from medical records (7, 8), whereas the utilisation of ARM techniques in identifying thyroid disease pathogenesis is still limited.

Collectively, this study sought to address the clinical challenge being revealing the pathogenesis of thyroid disease through the use of ARM techniques. The identified risk factors from data mining techniques will then be compared to those derived from the qualitative analysis to assess their reliability. Besides, this study involved two open-access datasets being evaluated with two ARM algorithms for knowledge discovery in obtaining impartial findings. To support the reproducibility of our work, the implementation procedure, code, and datasets can all be accessible through GitHub.

## 2 Background and Related Works

Thyroid disease is grabbing attention worldwide due to its rapidly increased diagnostic instances. Researchers tend to focus on improving the diagnostic efficiency of thyroid disease through machine learning techniques; nevertheless, the pathogenesis of thyroid disease has been considerably ignored by existing studies. With the objective to reveal the pathogenesis of thyroid disease through ARM applications, this section interprets the related literature studies around thyroid disease risk factors and the application of ARM in the medical domain.

### 2.1 Thyroid Disease Risk Factors

Thyroid disease instances are rising rapidly worldwide. Based on the statistics conducted by (2), there will be approximately 43,800 new cases being diagnosed with thyroid cancer in the United States in 2022. Although the diagnostic rates are continuously increasing, the cause of the disease remains unclear. Identifying the pathogenesis of thyroid disease can reduce morbidity and mortality rates since the risk factors associated with the disease can be potentially prevented.

There are extensive studies utilizing machine learning-driven techniques to help improve the diagnosis of thyroid disease with the surveillance of medical imaging, whereas studies deploying data mining-based approaches for investigating the cause of the disease are far limited. Furthermore, those existing studies majorly focused on determining the risk factors of thyroid disease through qualitative approaches. For instance (9), once conducted a systematic literature review incorporating 37 research studies for identifying the cause of thyroid disease. The results indicated that body mass index (BMI) was more associated with a high risk of thyroid cancer than diet habits and female reproductive relevant factors. Similarly (10), summarized existing literature studies and focused on investigating the association between diabetes and thyroid cancer. Their results indicated that the association was relatively weak, and further experiments were expected for confirmation. Collectively, through the qualitative analysis (i.e., particularly literature analysis), several other studies proposed some risk factors correlated with thyroid disease, such as vitamin D deficiency (11), diabetes (12), radiation (4), hormonal factors (13), and genetic factors (14).

Nevertheless, the limitation of the above studies is that they did not apply quantitative analytics to confirm those identified risk factors, leading to the restricted reliability of the findings. Therefore, many of those identified factors are still controversial in academia and clinical, as the factors were not empirically and experimentally evaluated. To better address those issues and verify the controversial risk factors, we bridged the literature gaps and utilized data mining techniques, specifically ARM algorithms, to investigate the hidden correlations of the risk factors with thyroid disease.

### 2.2 Association Rule Mining

Data mining is an effective tool for discovering potential meaningful and valuable knowledge from high-dimensional records (15). ARM, as a subset of data mining, intends to investigate the inherent correlations among different attributes given a database (16). ARM techniques demonstrated superior performance in knowledge discovery in many research domains (17, 18), leading to substantial developments for policy establishments, marketing analysis, and systems designs; more importantly, their potential applications in the medical domain are considerably inspiring.

Medical datasets are extensive in volume and high dimensional; mining knowledge from such heterogeneous, intricate, complex data is unattainable through a manual process. With the help of data mining techniques, we can effectively identify hidden associations among different diseases from those raw medical datasets for valuable knowledge discovery. Additionally, ARM techniques have been applied quite a few times in the medical domain for observing underlying correlations among different types of diseases. For instance (19), once investigated 1,560 patients with COVID-19 cases, their results found that the most common symptoms of COVID were fever, cough, pneumonia, and sore throat. Likewise (20), adopted the Z-Alizadeh Sani dataset (21) to identify the potential risk factors associated with cardiovascular disease, and they found that old age, typical chest pain, and obesity were three factors that are strongly related to the development of cardiovascular disease (22). utilized ARM algorithms to comprehend comorbidities with mental disorders. Their findings indicated that sleep disorders and digestive diseases were two comorbidities correlated with mental disorders.

Collectively, ARM has demonstrated superior performance in identifying underlying correlations among different types of diseases; thus, utilizing ARM to identify the risk factors associated with thyroid disease is feasible. Therefore, this research study sought to reveal the pathogenesis of thyroid disease by utilizing ARM techniques.

## 3 Research Methodology

Since knowledge extraction for thyroid disease pathogenesis is of primary research objective in this study, this section illustrates the research design, visualizes the designed framework indicating the flowchart of the experimental implementation, and explains the utilized ARM algorithms.

### 3.1 Proposed Framework

**Figure 1:**
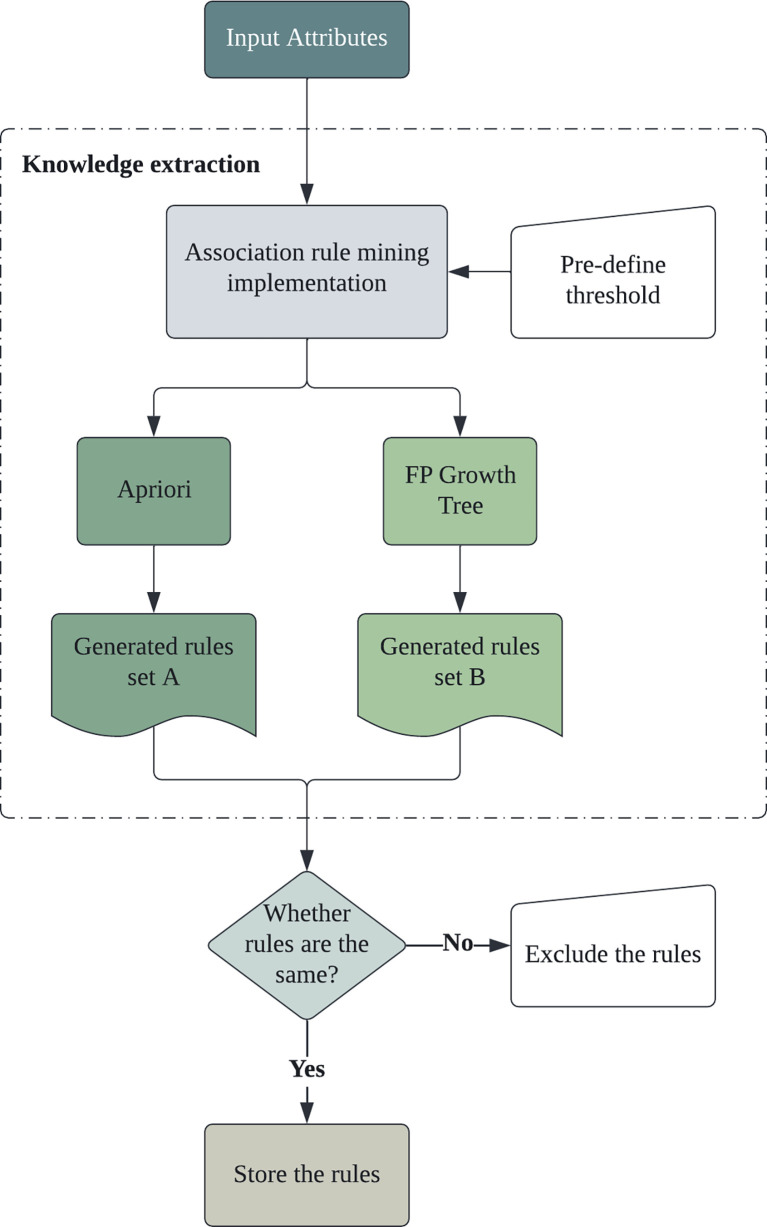
Thyroid disease pathogenesis knowledge extraction framework.

In order to better visualize the specific procedures of knowledge discovery for thyroid disease pathogenesis, **Figure 1** demonstrates the proposed framework, which outlines the ARM implementation process. Initially, the input of the framework would be the patient instance attributes, including potential categorical variables, such as demographic features, medical history, comorbidity, and examination results. Specifically, two open-access datasets were adopted for this research study so that the extracted rules from two independent data sources can be compared to ensure the fairness of the findings.

Those pre-processed input attributes will be fed into the ARM algorithms for rules extraction. In this study, we utilized two most classic ARM algorithms for knowledge discovery: the Apriori algorithm and the FP-Growth Tree algorithm, to obtain impartial results. The generated association rules from the aforementioned algorithms will be filtered based on the pre-defined thresholds of the support and confidence metrics. Moreover, the independently extracted association rule sets from two algorithms will be compared; the mutual rules will be selected and stored to be further interpreted. Those mutual rules are then considered the risk factors correlated to thyroid disease pathogenesis.

### 3.2 Association Rule Mining Algorithms

The ARM concept is to identify the probability occurrence of an antecedent A to a consequent C among multiple varied attributes (16), denoted as A ⇒ C indicating that if A exists, then C should co-exist in the database.

To verify the correlations between A and C, two metrics were generally utilized for evaluations: support and confidence values. Suppose we have a transaction database T, let T = {X_1,_ X_2_, …,X_N_} where X is the instance in T and N is the total number of instances in T. Within each instance X, there will be multiple attributes; thus, the instances can be further denoted as $X_{i}^{m}$ , in which m represents the multiple unique variables in each X_i_.

Based on these notations, the support values of each frequent itemsets are calculated through Eq. 1 which identifies the occurrence of the itemsets in the database. A large support value indicates that the itemset frequently shows in the database, whereas a low support value represents that the itemset is relatively infrequent and rarely presented in the database.


(1) $$Support=\frac{freq(A\Rightarrow C)}{N}$$

For the frequently appeared itemsets, the confidence values will also be calculated to identify their conditional probabilities through Eq. 2 to evaluate the confidence of an instance containing C and also contains C, or the other way around. A large confidence value indicates the strong associations between A and C, whereas a low confidence value denotes relatively weak correlations.


(2) $$\text{Confidence}=\frac{\text{freq}(\text{A}\Rightarrow \text{C})}{\text{freq}(\text{A})\;}$$

With pre-defined thresholds for support and confidence metrics, the generated rules can be mainly categorized into two types: frequent (i.e., rules that have large support and confidence values) and infrequent (i.e., rules that have low support and confidence values, or rules that have low support but high confidence) (23). This study only considers frequent rules for knowledge discovery; this is because medical knowledge discovery requires empirical evidence. Extract rules with high support and high confidence values guarantee the reliability of the knowledge since the rules are supported with a sufficient amount of popularity in place.

Furthermore, to better validate the generated rules, we incorporated two ARM algorithms for obtaining impartial results by extracting mutual rules, and the adopted algorithms are Apriori and FP-Growth Tree.

**The Apriori algorithm** was initially proposed by (24), which is the most classic ARM algorithm for data mining tasks. The Apriori algorithm can extract associations, frequent patterns, or even unexpected structures from unstructured transaction datasets. The algorithm follows a bottom-up approach to enumerate combinations of items in the database during iteration; thus, it is a table-based technique to list itemsets that appeared in the transaction. The algorithm is relatively straightforward, and detailed procedures are as follows:

- List all the unique items that appeared in the database
- Identify the support values for all unique items
- Prune the items which do not meet support threshold
- Loop through instances in the transaction dataset T
- In the loop, for each instance X_i_, enumerate all the combinations of the remaining items; go through each iteration until finish all instances in T
- Generate candidate itemsets based on enumerated combinations
- Calculate support values for all the candidate itemsets, and prune the ones below threshold
- Final rules are the frequent itemsets

**The FP-Growth Tree algorithm** was another well-known ARM technique that was proposed by (25). The FP-Tree Growth algorithm addressed the efficiency issue faced by the table-based Apriori algorithm and applied tree-based data structures to enhance the generation of frequent itemsets. Specifically, the FP-tree algorithm does not require candidate itemsets generation; with the tree structure, the pruning process is much more efficient. Since the tree only needs to be built once, making the rules extraction process faster when dealing with a relatively larger scale of dataset. And the detailed procedures are as follows:

- Identify unique items and calculate their support values in transaction T
- Write all the unique items in the descending order based on the support values
- Loop through each instance in T; for each instance X_i_, rewrite the attributes m based on their order
- Draw the FP-tree starting from “null” node; go through each instance X_i_ and draw the nodes, meanwhile record their frequency count
- Update the tree through each iteration and update the item frequency count until finishing all the instances in T
- Generate a conditional FP-tree if the support value for the node is larger than the threshold
- Generate frequent patterns based on the conditional FP-tree, and they are the final rules

We have utilized two independent datasets for the thyroid disease pathogenesis identification task by applying Apriori and FP-Growth to identify mutual risk factors.

## 4 Experimental Settings

With the objective to identify the most impartial findings, we included two algorithms and two datasets for knowledge discovery of thyroid disease pathogenesis. This section explains the utilized datasets and the parameters settings.

### 4.1 Dataset Descriptions

In order to ensure the reproducibility of our work and also to enlarge the potential implementations for different diseases, this study utilizes independent open-access datasets that were both retrieved from the UCI Machine Learning Repository (26). And the acquired datasets are explained as follows.

**Dataset-I Thyroid Disease Dataset** is an online thyroid dataset that Ross Quinlan provided in 1987 (26). The overall dataset consists of six databases, and this study utilizes the sick thyroid dataset for knowledge extraction. The sick dataset contains 2800 instances with 30 attributes related to the diagnosis of thyroid disease. After the data cleaning process, a total number of 2437 instances with 21 attributes were utilized in this research, and the selected attributes along with their descriptions are presented in **Table 1**.

**Table 1:** Dataset-I Thyroid Disease Dataset Selected Attributes.

Attributes	Descriptions	Values
Age	Age group intervals	20–30 (Age between 20 to 30)
		30–40 (Age between 30 to 40)
		40–50 (Age between 40 to 50)
		50–60 (Age between 50 to 60)
		60–70 (Age between 60 to 70)
		70–80 (Age between 70 to 80)
Sex	Gender groups	M=Male and F=female
On Thyroxine	On thyroxine status	f=False (Not on thyroxine)
		t=True (On thyroxine)
On anti-thyroid med	Anti-thyroid medication	f=False (Currently not on anti-thyroid med)
		t=True (Currently on anti-thyroid med)
Sick	Current sick status	f=False (Currently not sick)
		t=True (Currently sick)
Pregnant	Pregnant status	f=False (Currently not pregnant)
		t=True (Currently pregnant)
Thyroid surgery	Had thyroid surgery	f=False (Did not have thyroid surgery)
		t=True (Had thyroid surgery)
I131	Had I131 treatment	f=False (Did not have I131 treatment)
		t=True (Had I131 treatment)
Query hypothyroid	Hypothyroidism statue	f=False (Do not have hypothyroidism)
		t=True (Have hypothyroidism)
Query hyperthyroid	Hyperthyroidism status	f=False (Do not have hyperthyroidism)
		t=True (Have hyperthyroidism)
Lithium	Lithium status	f=False (Do not have Lithium)
		t=True (Have lithium)
Goiter	Goiter status	f=False (Do not have goiter)
		t=True (Have goiter)
Tumor	Tumor status	f=False (Do not have tumor)
		t=True (Have tumor)
Hypopituitary	Hypopituitary status	f=False (Do not have hypopituitary)
		t=True (Have hypopituitary)
Psych	Psych status	f=False (Do not have psych)
		t=True (Have psych)
TSH	TSH level intervals	TSH=Normal (0.27≤ TSH ≤4.2)
		TSH=Abnormal (Not within the normal range)
T3	T3 level intervals	T3=Normal (1.3≤ T3 ≤ 3.1)
		T3=Abnormal (Not within the normal range)
TT4	TT4 level intervals	TT4=Normal (62≤ TT4 ≤ 164)
		TT4=Abnormal (Not within the normal range)
T4U	T4U level intervals	T4U=Normal (0.7≤ T4U ≤ 1.8)
		T4U=Abnormal (Not within the normal range)
FTI	FTI level intervals	FTI=Normal (53≤ FTI ≤142)
		FTI=Abnormal (Not within the normal range)
Class	Thyroid disease status	Negative (Does not have thyroid disease)
		Positive (Has thyroid disease)

**Dataset-II Z-Alizadeh Sani Dataset** is a digital record dataset consisting of 303 instances; it was initially gathered for diagnosing cardiovascular disease (21). This dataset contains general demographic features from the collected patients, including their age, gender, weight, and height; besides, their smoking status and potential comorbidities were also included, making it suitable for detecting the risk factors correlated with thyroid disease since we can evaluate the potential associations between thyroid disease and other comorbidities. Specifically, 56 attributes were included in the dataset, whereas this study has included 15 features for data mining purposes. The selected features are demonstrated in **Table 2**.

**Table 2:** Dataset-II Z-Alizadeh Sani Dataset Selected Attributes.

Attributes	Descriptions	Values
Age	Age group intervals	≤=50 (Less than or equal to 50)
		>50 (Larger than 50)
Gender	Patient gender groups	M=Male, F=Female
DM	Diabetes Mellitus	0=No (Currently not have diabetes mellitus)
		1=Yes (Currently has diabetes mellitus)
HTN	Hypertension	0=No (Currently not have hypertension)
		1=Yes (Currently has hypertension)
Current Smoker	Current smoking status	0=No (Currently does not smoke)
		1=Yes (Currently smokes)
Previous Smoker	Previous smoking status	0=No (Did not smoke in the past)
		1=Yes (Used to smoke)
Obesity	Obesity disease status	N=No (Does not have obesity)
		Y=Yes (Has obesity)
CRF	Chronic Renal Failure	N=No (Does not have chronic renal failure)
		Y=Yes (Has chronic renal failure)
CVA	Cerebrovascular Accident	N=No (Did not have cerebrovascular accident)
		Y=Yes (Had cerebrovascular accident)
Airway Disease	Airway disease status	N=No (Does not have airway disease)
		Y=Yes (Has airway disease)
Edema	Edema status	0=No (Without edema)
		1=Yes (With edema)
Lung Rales	Lung rales status	N=No (Without lung rales)
		Y=Yes (With lung rales)
Dyspnea	Dyspnea status	N=No (Does not have dyspnea)
		Y=Yes (Has dyspnea)
Cardiovascular Disease	Cardiovascular disease status	N=No (Does not have CAD)
		Y=Yes (Has CAD)
Thyroid Disease	Thyroid disease status	N=Negative (Does not have thyroid disease)
		Y=Positive (Has thyroid disease)

### 4.2 Parameters Settings

Before inputting the attributes into the ARM algorithms, pre-processing of the input variables was conducted for both datasets. This study follows a rigorous mechanism for the experimental implementations. Specifically, the dataset has been processed with the following requirements:

- Instances with any missing values were removed.
- Numerical variables were discretized and assigned with distinct values.
- Different class of thyroid disease was split for knowledge extraction separately (i.e., healthy and sick groups were analyzed separately).

Furthermore, in order to better ensure the fairness and equity of the extracted knowledge from the two algorithms, the support and confidence thresholds were set to 0.6 and 0.9, respectively, for both datasets during the association rule mining implementation stage.

## 5 Results

Following the procedures demonstrated in the proposed framework, the rules extracted by the Apriori and the FP-Tree algorithms were compared and analyzed. Based on our analysis, the Apriori and the FP-Growth Tree algorithms produced similar results. Therefore, only the mutual rules from both algorithms were selected and exhibited in this study. And this section displays the extracted mutual rules.

### 5.1 Dataset-I Results

Based on the analysis of the 21 features from the sick thyroid dataset, **Table 3** exhibits the extracted rules for both healthy and sick groups of patients. Specifically, in the healthy group, a history of I131 treatment plays a significant role in determining the current status of thyroid disease. When the target patients have no history of I131 treatment or hypopituitary, they are less likely to be diagnosed with thyroid disease with a support value of 0.98 and a confidence value of 1.00. Besides, when the instance has no history of I131 treatment or hyperthyroid, he/she is very likely to be in the healthy group with a support of 0.92 and confidence of 1.00.

**Table 3:** Dataset-I Thyroid Disease Dataset Results.

Groups	Antecedents		Consequent	Support	Confidence
Healthy	I131=False, Hypopituitary = False	⇒	Negative	0.98	1.00
	I131= False, Lithuim = False	⇒	Negative	0.98	1.00
	I131= False, On_antithyroid_med=False	⇒	Negative	0.97	1.00
	I131=False, Query_hyperthyroid=False	⇒	Negative	0.92	1.00
	I131= False, On_thyroxine=False	⇒	Negative	0.86	1.00
Sick	T3=Abnormal	⇒	Positive	0.88	1.00
	T3=Abnormal, Goiter=False	⇒	Positive	0.87	1.00
	T3=Abnormal, Query_hyperthyroid=False	⇒	Positive	0.85	1.00
	Female, T3=Abnormal	⇒	Positive	0.80	1.00
	Female, Thyroid_surgery=False	⇒	Positive	0.61	1.00

In the sick group, Triiodothyronine (T3) is a crucial factor related to thyroid disease development. When the instance has an abnormal range of T3 level, the patient is very likely to be diagnosed with thyroid disease; this rule has a support value of 0.88 and a confidence value of 1.00. Besides, when the patient is female with an abnormal T3 range, she is at high risk of being diagnosed with thyroid disease with a support of 0.80 and confidence of 1.00. Therefore, gender, a history of I131 treatment, and T3 range are three risk factors that can be used for determining current thyroid disease existence.

### 5.2 Dataset-II Results

The Z-Alizadeh Sani dataset provides more comprehensive features for identifying the potential risk factors correlated with thyroid disease. Besides the basic demographical features, many comorbidities were also involved. **Table 4** demonstrates the extracted rules with this dataset. For the healthy group, patients without a history of chronic renal failure (CRF) and cerebrovascular accident (CVA) are very likely to be grouped into the negative class with a support of 0.97, suggesting that they are less likely to be diagnosed with thyroid disease. Besides CRF, age is another factor contributing relatively strongly to the diagnosis of thyroid disease.

**Table 4:** Dataset-II Z-Alizadeh Dataset Results.

Groups	Antecedents		Consequent	Support	Confidence
Healthy	CRF=False,CVA=False	⇒	Negative	0.97	1.00
	CRF=False,Ex_smoker=False	⇒	Negative	0.95	1.00
	CRF=False,Age>50,AD=False	⇒	Negative	0.71	1.00
	Age>50,Edema=False,LR=False	⇒	Negative	0.70	1.00
	AD=False,Diabetes_mellitus=False	⇒	Negative	0.68	1.00
Sick	Hypertension=True	⇒	Positive	0.71	1.00
	Obesity=True	⇒	Positive	0.71	1.00
	Hypertension=True,CRF=False	⇒	Positive	0.71	1.00
	Obesity=True,Ex_smoker=False	⇒	Positive	0.71	1.00

Whereas for the sick group, few comorbidities demonstrated the associations with the existence of thyroid disease. In particular, when the patient has hypertension, he is very likely to be diagnosed with thyroid disease; this rule has a support value of 0.71 and a confidence value of 1.00. Similarly, if the patient has obesity, then he/she is also at high risk of developing thyroid disease with the same support and confidence values. Moreover, females are likely to develop thyroid disease, yet, smoking status did not play a critical role here. Therefore, age, gender, hypertension, and obesity seem to be considered influential in developing thyroid disease.

## 6 Discussion

With the continuous growth of thyroid disease instances, scholars were dedicated to improving the diagnostic efficiency with the help of machine learning techniques (27, 28), whereas the cause of the disease remains under-researched. Although enhancing the diagnostic performance for thyroid disease is imperative, investigating the pathogenesis is of primary research goal since the risk factors can be prevented to potentially mitigate morbidity and mortality rates brought by the disease. ARM techniques have been proven to be effective in knowledge extraction for identifying potential comorbidity (6, 22, 29), whereas their implementation on thyroid disease pathogenesis is still absent. This study then bridges the literature gap and adopts two ARM algorithms to identify risk factors correlated with thyroid disease.

Based on our analysis of the extracted rules from two distinct datasets, the results confirmed that gender and age are two factors strongly related to thyroid disease. Specifically, the consensus was established that females are more likely to be diagnosed with thyroid disease than males, and this finding aligns with existing studies proposed by (30, 31). Gender disparity in thyroid disease is a long-established topic, and the reason behind the higher diagnostic probabilities in females might be due to the hormonal effects brought by pregnancy or pubertal development, which were considered sensitive to young females (13, 32). The regular health examination mechanism might also be a hypothesis contributing to the enhanced thyroid disease diagnostic rates.

This study also identifies that hypertension and obesity are two leading factors, and this finding is in accordance with (33) and (12), suggesting that the existence of the underlying diseases might increase the development of thyroiddisease risk (34). presented that hypertension is closely related to overt hypothyroidism. The underlying mechanism could be edema caused by hypothyroidism, which heavily burdens the circulatory system in the body. This potentially leads to hypertension, especially the increase in diastolic blood pressure (35). once also stated that the diastolic blood pressure of hypothyroidism patients was three times more among others. Additionally, the excessive T3 level leads to metabolic and hemodynamic changes, which potentially cause an increased cardiac output and hypertension (34). More importantly, patients with hyperthyroidism caused by Graves’ disease or multi-nodular goitre may also present pulmonary hypertension (34). Nevertheless, studies addressing the correlation between hypertension and thyroid nodules are still absent, and further works should be presented to bridge this gap. Accordingly, practical applications can be made in the clinical field: if identifying increased blood pressure, it is suggested to evaluate thyroid functions. This helps reduce the health effects of abnormal thyroid functioning, such as problems related to high blood pressure, by adjusting thyroid hormones.

EmeraldOur study shows that thyroid disease is more likely to be developed among obese patients, and this finding is consistent with those reported in (36–38). Body mass index (BMI) is a metric for evaluating obesity, and patients with BMI values higher than 30kg/m^2^ are regarded as obese (39). Several studies have already determined a positive association between high BMI and a higher risk of thyroid cancer (40–42). In contrast, the association between obesity and thyroid cancer is yet to be explicitly outlined (43). indicated obesity is a factor that induces overt hypothyroidism, sub-clinical hypothyroidism, and Hashimoto’s thyroiditis. The underlying explanation could be that obese patients have enhanced thyroid autoimmunity risks, leading to the increased possibility of thyroid disease development (44). also suggested that abnormal thyroid functions are associated with high BMI, contributing to possible correlations between obesity and thyroid disease. Moreover, obesity is a vital factor for autoimmune inflammatory diseases like diabetes (43). Emerald (45) and (46) suggested that diabetes might also have associations with an increased risk of thyroid disease; however, it was controversial with our results. We suggest that further clinical assessments should be involved to regularly screen for thyroid functions in hypertensive and obese populations.

Collectively, this research utilizes two ARM algorithms on two independent datasets to extract risk factors correlated with thyroid disease. Although the proposed method can be adapted to different diseases, there are still a few limitations in this study, many of which correlate with the sample data. The sample size used in this work for identifying the pathogenesis is relatively limited. Moreover, the incorporated features in this work are relatively narrow. Many possible factors were not evaluated since they were not included in the selected datasets, such as hormonal factors, gene heredity, vitamin D deficiency, or family history. In future works, we sought to include more sample instances containing more distinct factors for a comprehensive evaluation of the pathogenesis of thyroid disease. Possible feature selection algorithms will need to be involved in comparing the ARM techniques. Additionally, machine learning-based classification algorithms will be incorporated with the feature selection algorithms to make predictions for detecting thyroid disease.

## 7 Conclusion

Despite the increased diagnostic rates of thyroid disease, its pathogenesis remains unclear. This study utilizes two association rule mining algorithms for knowledge extraction to reveal the potential risk factors correlated with thyroid disease.

The findings suggest that gender, a history of I131 treatment, T3 level, hypertension, and obesity are critical factors associated with thyroid disease development. This research study emphasizes the contributions made to society; by identifying the correlated risk factors, thyroid disease’s mortality and morbidity rates can be considerably mitigated.

## Data Availability Statement

The original contributions presented in the study are included in the article/supplementary material. Further inquiries can be directed to the corresponding author.

## Author Contributions

All authors contributed to the study conception and design. Material preparation, data collection and analysis were performed by FL and XZ. The first draft of the manuscript was written by XZ and all authors commented on previous versions of the manuscript. All authors contributed to the article and approved the submitted version.

## Conflict of Interest

The authors declare that the research was conducted in the absence of any commercial or financial relationships that could be construed as a potential conflict of interest.

## Publisher’s Note

All claims expressed in this article are solely those of the authors and do not necessarily represent those of their affiliated organizations, or those of the publisher, the editors and the reviewers. Any product that may be evaluated in this article, or claim that may be made by its manufacturer, is not guaranteed or endorsed by the publisher.
